# EFFECTIVENESS OF AN AGING-RELATED PREPARATION EDUCATION PROGRAM FOR OLDER CHINESE MIGRANTS IN JAPAN

**DOI:** 10.1093/geroni/igae098.3156

**Published:** 2024-12-31

**Authors:** Li Yao, Harue Masaki

**Affiliations:** Chiba University, Chiba, Chiba, Japan; Chiba University, Chiba, Chiba, Japan

## Abstract

**Introduction and objectives:**

To resolve the long-term care issues of older Chinese migrants in Japan, we developed an aging-related education program focused on preparation for long-term care consisting of six modules with lectures and discussions through real-time videoconferences. This study aimed to evaluate the effectiveness of this developed education program among older Chinese in Japan.

**Methods:**

Older Chinese people and their family members living at home independently in the Tokyo and Kansai areas were recruited. The before-after study was conducted to collect the data from the Preparation for Future Care Needs Scale-14 (PFCN-14) and Attitudes to Aging Questionnaire (AAQ). Data was analyzed by descriptive statistics and the Friedman non-parametric repeated measures ANOVA test.

**Results:**

Twenty-two participants with an average age of 73.0 (SD= ±7.18) years completed all questionnaires, of which 14 (63.6%) were female. The median (IQR) PFCN-14 total scores before intervention, after intervention, and one month after intervention were 40.00 (7)%, 46.50 (13)%, and 48.50 (8)% (χ²=18.644, P< 0.001). There were significant differences between before and after intervention (P=0.016) and between before and one month after intervention (P< 0.001). The median (IQR) AAQ total scores were 82.00 (23)%, 80.50 (22)%, and 79.00 (21)%, respectively, with no significant differences (χ²=2.533, P=0.279).

**Conclusion:**

This education program improved participants’ status of preparing for long-term care by promoting awareness, decision-making, and concrete planning actions. It can be used to improve health literacy and the autonomy of later life for older Chinese migrants in Japan.

